# μABC: a systematic microsecond molecular dynamics study of tetranucleotide sequence effects in B-DNA

**DOI:** 10.1093/nar/gku855

**Published:** 2014-09-26

**Authors:** Marco Pasi, John H. Maddocks, David Beveridge, Thomas C. Bishop, David A. Case, Thomas Cheatham, Pablo D. Dans, B. Jayaram, Filip Lankas, Charles Laughton, Jonathan Mitchell, Roman Osman, Modesto Orozco, Alberto Pérez, Daiva Petkevičiūtė, Nada Spackova, Jiri Sponer, Krystyna Zakrzewska, Richard Lavery

**Affiliations:** 1 Section de Mathématiques, Swiss Federal Institute of Technology (EPFL), CH-1015 Lausanne, Switzerland; 2 Department of Chemistry, Wesleyan University, Middletown, CT 06459, USA; 3 Departments of Chemistry and Physics, Louisiana Tech University, Ruston, LA 71270, USA; 4 BioMaPS Institute and Deptartment of Chemistry and Chemical Biology, Rutgers University, 610 Taylor Road, Piscataway, NJ 08854-8087, USA; 5 Department of Medicinal Chemistry, University of Utah, Skaggs 307, Salt Lake City, UT 84112, USA; 6 Joint BSC-CRG-IRB Program on Computational Biology, Institute of Research in Biomedicine, Parc Científic de Barcelona, Josep Samitier 1-5, Barcelona 08028, Spain; 7 Barcelona Supercomputing Centre, Jordi Girona 31, Edifici Torre Girona, Barcelona 08034, Spain; 8 Departament de Bioquímica, Facultat de Biología, Avgda Diagonal 647, Barcelona 08028, Spain; 9 Department of Chemistry, Indian Institute of Technology, Hauz Khas, New Delhi 110016, India; 10 Institute of Organic Chemistry and Biochemistry, Academy of Sciences of the Czech Republic, Flemingovo nam. 2, 166 10 Praha 6, Czech Republic; 11 School of Pharmacy and Centre for Biomolecular Sciences, University of Nottingham, NG7 2RD, UK; 12 Department of Structural and Chemical Biology, Mount Sinai School of Medicine, New York, NY 10029, USA; 13 Institute of Biophysics, Academy of Sciences of the Czech Republic, Kralovopolska 135, 612 65 Brno, Czech Republic; 14 CEITEC—Central European Institute of Technology, Masaryk University, Campus Bohunice, Kamenice 5, 625 00 Brno, Czech Republic; 15 Bases Moléculaires et Structurales des Systèmes Infectieux, CNRS UMR 5086/Université Lyon I, IBCP, 7 Passage du Vercors, 69367 Lyon, France

## Abstract

We present the results of microsecond molecular dynamics simulations carried out by the ABC group of laboratories on a set of B-DNA oligomers containing the 136 distinct tetranucleotide base sequences. We demonstrate that the resulting trajectories have extensively sampled the conformational space accessible to B-DNA at room temperature. We confirm that base sequence effects depend strongly not only on the specific base pair step, but also on the specific base pairs that flank each step. Beyond sequence effects on average helical parameters and conformational fluctuations, we also identify tetranucleotide sequences that oscillate between several distinct conformational substates. By analyzing the conformation of the phosphodiester backbones, it is possible to understand for which sequences these substates will arise, and what impact they will have on specific helical parameters.

## INTRODUCTION

Once the first single crystal structure of a DNA oligomer was obtained (1,2), it became clear that base sequence could have a significant impact on structure, even within a single conformational family, such as B-DNA. This finding was of major interest since DNA cannot express its genetic message, be replicated, repaired or packaged within the cell without interacting with proteins. Sequence-dependent conformational variations can be expected to play a significant role in how proteins interact with the double helix, and notably in determining how specifically binding proteins recognize their target sites (3–7). Such ‘indirect recognition’ processes have now been confirmed in a wide variety of protein–DNA interactions. Similar effects can be expected to play a role in drug–DNA interactions (which include many important anticancer agents) (8) and may also be important in designing superstructures built from DNA in the field now known as DNA origami (9,10).

Accumulated structural data on DNA oligomers have shown that some sequences have markedly different behaviors from others, but unfortunately there are still not enough experimental data on isolated, unmodified B-DNA oligomers to be able to generate a complete picture of sequence-dependent effects. It has, however, become clear that individual dinucleotide steps can have different conformational statistics as a function of their tetranucleotide sequence contexts (i.e. their 5′ and 3′ flanking base pairs). As previously explained (11), this does not imply that the sequence dependence of a coarse-grain model of the conformational free energy of an oligomer need depend upon more than the overlapping dinucleotide sequence context, and such models can be rather accurate (12). However, it does mean that the conformational characteristics of the 10 distinct dinucleotide steps embedded in all possible flanking base pairs, i.e. the conformational statistics of at least the 136 distinct tetranucleotides (AAAA, AAAT, AAAC, etc.), must be examined. Interrogating the Nucleic Acid Database (13) for isolated, unmodified B-DNA crystal structures resolved to at least 2.5 Å shows that while some tetranucleotides are strongly represented (e.g. CGAA, GAAT, etc.), in only a very few cases are there enough data to guarantee the statistical significance of any derived average value (14), and half of the tetranucleotides are completely absent (data from May 2014).

Given this state of affairs, a group of laboratories came together in 2002 with the aim of using molecular simulations to create a balanced structural database at the tetranucleotide level that would provide a guide to sequence effects both on the structure and on the dynamic properties of the B-DNA double helix. This group, termed the Ascona B-DNA Consortium (or ABC for short), began by designing an approach for studying the 136 distinct tetranucleotides with the least possible computational cost. The answer was to design oligomers of the type gc-*CD-ABCD-ABCD-ABCD*-gc, each containing 3.5 tetranucleotide repeats (shown in italics) placed between GC termini (shown in lower case, chosen to reduce ‘fraying’), leading to a total of 18 base pairs. This choice meant that all tetranucleotides could be packed into only 39 oligomers (since, in the best cases, one oligomer would contain four different tetranucleotides: *ABCD, BCDA, CDAB*, and *DABC*). In addition, the repeating sequences made it possible to test for convergence by comparing multiple instances of equivalent tetranucleotides within a single oligomer.

The ABC laboratories have already completed two series of computations using the AMBER suite of programs (15,16) to model DNA oligomers in their natural aqueous environment (explicitly representing water molecules and sufficient ions to reach a physiological salt concentration). The first series (17,18), limited to 15 ns trajectories for each oligomer, showed surprisingly important sequence-dependent changes in structure, but also detected problems with the force field that led to the accumulation of unusual backbone conformations and increasing helical deformations. This problem was solved by a limited modification of the backbone parameters (19) leading to improved agreement with experiment (20–23) and a second series of simulations with 50–100 ns trajectories for each oligomer. This study brought to light the existence of oscillations between conformational substates for certain tetranucleotides, where significant changes in helical parameters were coupled to changes in backbone torsions (24) (see also (14)). Given the timescale of these oscillations, it became clear that longer trajectories were necessary to correctly sample the large conformational space available to B-DNA at room temperature. The availability of better computational resources made this aim possible, and we are now able to present the results of simulations that have been extended to at least one microsecond for each of the 39 oligomers. Even with today's computational facilities, this represents a significant investment leading to a total of roughly 60 μs of trajectories and 9 Tb of data, including more than 35 million conformational snapshots.

As part of the ABC project, we have also developed tools (notably Curves+ and Canal) for analyzing not only individual nucleic acid structures, but also long molecular dynamics trajectories (25,26). Several refinements have been made to these tools as a result of the study presented here and these are discussed in the Materials and Methods section. A further development involves analyzing ion distributions using curvilinear helicoidal coordinates (27). Its application to the present set of trajectories will be the subject of a separate publication.

The results from this new series of simulations represent a significant step toward the main ABC goal, namely understanding base-sequence effects on B-DNA. First, the trajectories sample the B-DNA conformational basin much more thoroughly than any previous work. They confirm that many sequences occupy more than one conformational substate at room temperature, and that these substates can have dramatically different helical parameters. They show that base sequence modifies DNA fluctuations, but in a selective manner, often affecting only one, or a small subset, of helical parameters. Lastly, building on recent work studying the conformational behavior of specifically the CpG dinucleotide step (28), they provide better understanding of the mechanisms that lead to multiple substates and clarify in which cases substates will arise. Taken together, these microsecond trajectories not only provide insight into the conformational mechanics of B-DNA, but also constitute a valuable resource for developing coarse-grain models of double-helical DNA.

## MATERIALS AND METHODS

The results discussed in this article are based on molecular dynamics trajectories for 39 double-stranded B-DNA oligomers, each containing 18 base pairs. The sequence of each oligomer is constructed in the same way: 5′-gc-*CD-ABCD-ABCD-ABCD*-gc-3′, where upper case letters indicate sequences that vary between oligomers and lower case letters indicate fixed sequences (dashes have been added for clarity). Every oligomer contains a four base pair sequence, *ABCD*, that is repeated three and a half times. This sequence is used as the name for each oligomer. The full list of the 39 oligomers is given in Supplementary Table S1. Taken together, these oligomers contain all 136 distinct tetranucleotide sequences as shown in the table.

Molecular dynamics simulations on the oligomers were carried out with periodic boundary conditions within a truncated octahedral cell using the AMBER suite of programs (15,16) with the parmbsc0 modifications (19) to the parm99 force field (16,29), Dang parameters (30) for the ions and SPC/E water (31). Each oligomer was neutralized with 34 potassium ions and then an appropriate number of K^+^Cl^−^ ion pairs were added to reach a salt concentration of 150 mM. Ions were initially placed at random within the simulation cell, but at least 5 Å from DNA and at least 3.5 Å from one another. The complex was then solvated with a layer of water at least 10 Å thick. A typical simulation involved ∼11 500 water molecules and 37 000 atoms in total. Electrostatic interactions were treated using the particle mesh Ewald method (32) with a real-space cutoff of 9 Å and cubic B-spline interpolation onto the charge grid with a spacing of 1 Å. Lennard–Jones interactions were truncated at 9 Å and the pair list was built with a buffer region and a triggered list update whenever a particle moved more than 0.5 Å from the previous update.

During the preceding ABC project, at least 50 ns trajectories were obtained for each of the 39 oligomers (24). This involved constructing each oligomer in a canonical B-DNA conformation, equilibrating the system by energy minimization of the solvent, and then of the solute and solvent together, followed by a slow thermalization, following the protocol described earlier (17,18,24). Simulations were carried out using an NPT ensemble, using the Berendsen algorithm (33) to control temperature and pressure, with a coupling constant of 5 ps for both parameters. All chemical bonds involving hydrogen atoms were restrained using SHAKE (34), allowing for stable simulations with a 2 fs time step. Center of mass motion was removed every 5000 steps to avoid kinetic energy building up in translational motion (35) and to keep the solute centered in the simulation cell. The end of the 50 ns simulations was used as the starting point for the current ABC project where each trajectory was extended to 1 μs. In the present work, we treat the first 100 ns of the trajectories as an extended equilibration period and analyze only the remaining 900 ns of simulation. Conformational snapshots were saved from each trajectory at 1 ps intervals, leading to a database of 35 million snapshots which represents 9 Tb of data including solvent and 300 Gb if the water molecules are removed.

The first stage of conformational analysis was performed using Curves+ (25), which provides a full set of helical, backbone and groove geometry parameters. Curves+ uses the commonly agreed ‘Tsukuba’ reference frame to describe each base (36) and respects the Cambridge convention for the names and signs of all helical parameters (37). Parameters are grouped into five sets: (i) intra-base pair, or intra-BP for short (shear, stretch, stagger, buckle, propeller, opening); (ii) BP-axis (Xdisp, Ydisp, inclination and tip); (iii) inter-BP (shift, slide, rise, tilt, roll, twist); (iv) backbone (in the 5′→3′ direction for each nucleotide, α P-O5′, β O5′-C5′, γ C5′-C4′, δ C4′-C3′, ϵ C3′-O3′, ζ O3′-P, the glycosidic angle χ C1′-N1/N9 and the sugar pucker phase and amplitude); (v) groove (minor and major groove widths and depths). Note that the rise and twist discussed in this article are the parameters derived from the matrix transformation between two base pair reference frames. Curves+ also calculates these parameters as a translation and a rotation around the helical axis, but, in the case of B-DNA, the difference between the two sets of parameters is not significant. The reader is referred to a previous publication for further details (25) (and, notably, to Supplementary Figure S1 of the corresponding supplementary material which illustrates all the helical parameters).

We also remark that when we consider the conformation or the dynamics of a given sequence fragment, we only discuss the conformational parameters connected with the center of the fragment. Thus, if the fragment contains an odd number of base pairs, we discuss the central base pair in terms of intra-BP, BP-axis and groove dimensions, as well as those parts of the backbone directly associated with this base pair (glycosidic torsions and sugar pucker). If the fragment contains an even number of base pairs, we discuss the central base pair step in terms of the inter-BP parameters and the backbone torsions integral to the base pair step (in the 5′-3′ direction for each strand: ϵ, ζ, α, β and γ). All Curves+ parameters are output in a file containing a single record for each snapshot in each oligomer. These files were then analyzed with the program Canal to obtain statistical data on all parameters, as well as time series, parameter distributions (in the form of histograms) and to search for correlations between parameters. Canal is used here to analyze individual trajectories and to make cumulative analyses over many trajectories. The present versions of Curves+, Canal and other related software are available at http://bisi.ibcp.fr/tools/curves_plus/.

Note that earlier versions of Canal calculated cross-correlations using standard algorithms for linear variables. These can be applied to angles if their range is small. However, for long simulations of DNA it is necessary to use formulae adapted to circular variables for backbone dihedrals (and, for security, rotational helical parameters). There exist a number of methods to estimate circular correlation (see for example (38–40), and references therein). Since we need to discriminate positive and negative correlation, and require values that are consistent with the Pearson correlation coefficients used for linear variables, we have excluded rank-based approaches. We chose the T-linear association measure $\hat \rho _T$ proposed by Fisher (38) that is robust and can be coded as a single pass algorithm (in common with the other analyses in Canal), which is essential for treating long trajectories. For these calculations, linear variables *x*, measured in angstroms, are converted to angles $\theta _x = \tan ^{ - 1} \frac{x}{5}$, where, as discussed for example in (11), 5 Å is an appropriate length scaling between angular and linear variables. Circular–circular and circular–linear correlation measures calculated in this way are very close to the Pearson correlation coefficient when its use is justified for angular variables, namely when the angles are distributed over a narrow range.

### Remarks on nomenclature and Watson–Crick symmetry

Following the conventions established in our prior work (17,18,24), sequence fragments in double-stranded DNA are always written in the 5′-3′ direction as a simple string of letters (e.g. the tetranucleotide ACGT, or the dinucleotide step CG) along one of the two backbones, which we designate as the Watson strand. As we assume canonical base pairing throughout, the sequence along the Crick strand is immediately implied, and is again always given in the (anti-parallel) 5′-3′ direction. Note that for the Curves+ (25) helical parameters adopted here, and for any configuration, changing the choice of the Watson strand re-orders the variables and switches the signs of four helical variables: shift, tilt, shear and buckle, while leaving the other eight intra- and inter-helical parameters unchanged. We exploit this point later. If we want to refer to dinucleotide step helical parameters within a longer sequence, we use an underline to indicate the step in question (e.g. ACGT). The letter R is used to indicate a purine, i.e. adenine A or guanine G, while Y indicates a pyrimidine, cytosine C or thymine T, and X indicates either R or Y, i.e. any of the four possible bases. When we refer to the base pairs flanking a dinucleotide step, we separate them by two dots (e.g. R..Y).

Note that there are three distinct dinucleotide steps in the pyrimidine/purine sequence alphabet, namely two self-symmetric steps YR and RY, and the non-symmetric RR/YY step. Similarly with the full (A,T,C,G) base alphabet, there are 10 distinct dinucleotide steps, four self-symmetric steps (AT, TA, CG, and GC) and six pairs of non-symmetric steps (AA/TT, GG/CC, AG/CT, GA/TC, GT/AC, and TG/CA). Following our previous work, we choose to label the 10 distinct steps as AA, AT, TA, GG, GC, CG, GA, AG, GT, and TG (by convention using the label with the most purines, and, if this is balanced, by choosing G in preference to A). Note that the choice of names for the six non-symmetric dinucleotides privileges one of the two junction backbones, which we will accordingly describe as the Watson backbone.

The analogous counts for tetranucleotides are slightly less familiar. With a purine/pyrimidine alphabet there are 10 physically distinct tetranucleotides, four self-symmetric (e.g. YRYR) and six non-symmetric pairs (e.g. RRYR/YRYY). With the full (A,T,C,G) alphabet there are 136 distinct tetranucleotides, 16 self-symmetric and 120 non-symmetric pairs. The choice of naming of the non-symmetric pairs is detailed in Supplementary Table S1. These tetranucleotides can also be grouped in terms of their central dinucleotide steps. For each of the four self-symmetric dinucleotide steps, there are 10 distinct choices of the flanking base pairs (four of the form R..R and three of the R..Y and Y..R forms). For each of the six non-symmetric dinucleotide steps, there are 16 choices of flanking base pairs (four in each of the R..R, R..Y, Y..R, and Y..Y families). In the following, unless otherwise indicated, information on the 136 distinct tetranucleotides is always extracted from the centermost occurrence of each tetranucleotide in the relevant oligomer simulation (e.g. TACG refers to positions 8, 9, 10 and 11 of oligomer 5′-gc-GTACGTACGTACGT-gc-3′, and so on).

## RESULTS AND DISCUSSION

### Convergence and sequence-averaged conformations

We begin by discussing the convergence of the microsecond simulations. As explained above, the initial design of the ABC oligomers was chosen so that the 136 distinct tetranucleotide sequences could be packed into a minimum number of oligomers. This choice has the additional advantage that each tetranucleotide occurs several times within each oligomer and thus differences between the average conformations of the repeating sequence elements can be used as a test for conformational convergence. The results shown in Supplementary Figures S1 and S2 compare the helical and backbone parameters for two tetranucleotides with identical sequences near the center of the oligomers (positions 6–9 and 10–13). The differences between the average parameters vary between oligomers, but even in the worst cases are within 0.25 Å for translational helical parameters, within 2° for rotational helical parameters, and within 5° for backbone dihedrals and sugar puckers and, in most cases, they are less than half these values.

Next we consider the time convergence of the simulations. Supplementary Figures S3 and S4 present the results for two oligomers by comparing average helical and backbone parameters calculated for the first or second half of a trajectory with those calculated over the full trajectory. The results again show good convergence with small differences between the average parameters. The worst cases show differences limited to less than 0.1 Å for translational helical parameters, less than 2° for rotational helical parameters, and 3° for backbone dihedrals and sugar puckers, with the exception of ϵ (C3′-O3′) and ζ (O3′-P) which can, in rare cases, reach 8° (see the discussion below). We remark in passing that a preliminary analysis of ion distributions for two of this set of ABC trajectories also shows that these distributions are stabilized within the microsecond timescale (27).

These results suggest that the microsecond trajectories are well converged with respect to the conformational space accessible to B-DNA oligomers at room temperature. As discussed below, this timescale allows for significant conformational fluctuations in both helical and backbone parameters, including the temporary loss of base pairing, particularly for the two or three base pairs at the ends of the oligomers, but also (transiently) for the more central base pairs. As we will discuss below, many of the oligomers exhibit multiple conformational substates involving specific conformational transitions. However, if we average over all the 39 oligomers, the overall structure resembles what is expected for a canonical B-DNA double helix as shown in Table 1 (where the results are averaged over the central base pair steps of the 136 distinct tetranucleotide sequences). These results are also very similar to the averages obtained from our earlier work with 50 ns trajectories (24). The average B-DNA structure shows relatively little deformation with respect to planar Watson–Crick base pairs, apart from an 11° propeller twist. The base pairs are on average inclined by 7° to the helical axis, shifted 1.5 Å toward the minor groove and show a small negative slide and positive roll. We remark that the average helical twist (32.5°), although improved by the BSC0 corrections to the force field (19), is 1°–2° lower than the estimated average solution value.

**Table 1.**
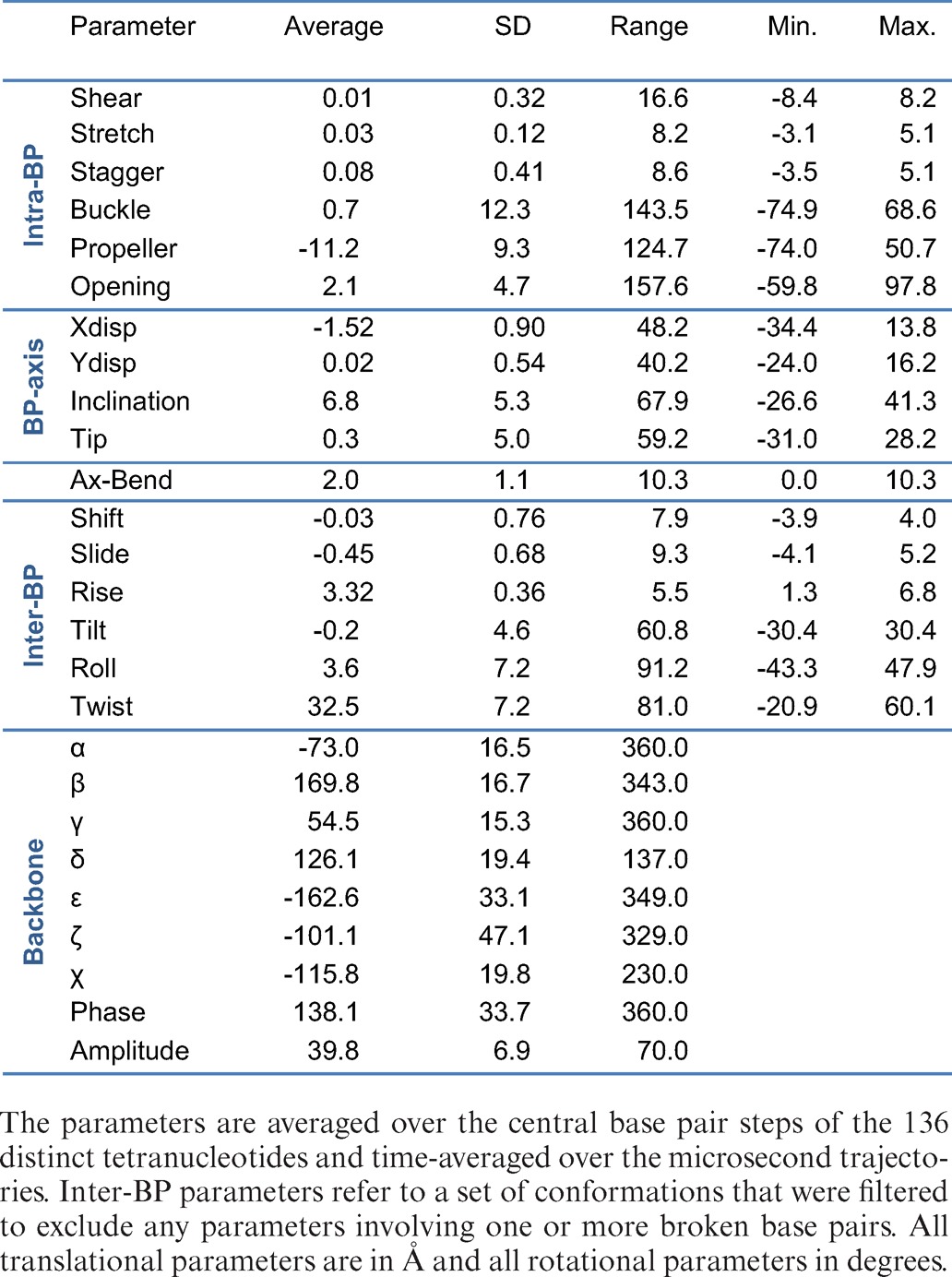
Sequence-averaged conformational parameters and fluctuations

Concerning conformational fluctuations, the columns minimum, maximum and range (the translational or angular extent covered by each parameter) of Table 1 show the impressive flexibility of DNA on the microsecond timescale. Note that, to avoid unrealistic values for the inter-BP parameters, we have filtered the snapshots to remove any such parameters involving base pairs with one or more broken Watson–Crick hydrogen bonds (i.e. with a distance between heavy atoms greater than 3.5 Å). Despite this precaution, twist can vary from −20° to 60° and rise from 1.3 Å to 6.8 Å. The largest values of rise correspond to the spontaneous generation of a potential intercalation site within the double helix (in line with the fluctuations observed in single-molecule experiments on DNA (41)). Similarly, spontaneous kinks occur at single base pair steps both toward the minor and major grooves, with extreme roll angles of −43° and 48°, respectively, creating strong bends in the helical axis. The fact that base pairs indeed transiently break within the central tetranucleotide of these 18 bp oligomers (42) can be seen from the unfiltered intra-BP parameters, where opening ranges from −60° (opening into the minor groove) to 98° (opening into the major groove). We can also see that the backbone angles (with the exception of δ that is constrained by the sugar ring pucker and χ that is constrained by stacked base orientations) cover almost their full angular ranges. Lastly, large fluctuations are seen in both groove width and depth. (Note that width measurements are based on distance between spline curves through the phosphorus atoms, reduced by 2 × 2.9 Å to allow for the size of the phosphate groups. Depth measurements involve the long axis of the base pairs and are reduced by 3.5 Å to allow for the half-width of the base pairs.)

### Tetranucleotide sequence effects on inter-base pair helical parameters

We now turn to the effects of base sequence on B-DNA conformation. Both available experimental structures of B-DNA (43), and our earlier simulations (17,18,24), show that analyzing the effect of sequence on parameter statistics in terms of only the overlapping dinucleotide steps is not sufficiently refined, because many dinucleotides show markedly different conformations as a function of their flanking base pairs. The oligomers chosen for the ABC study allow such effects to be observed by calculating average parameters for the 10 distinct base pair steps embedded in each of the possible tetranucleotide flanking sequence environments.

**Figure 1.**
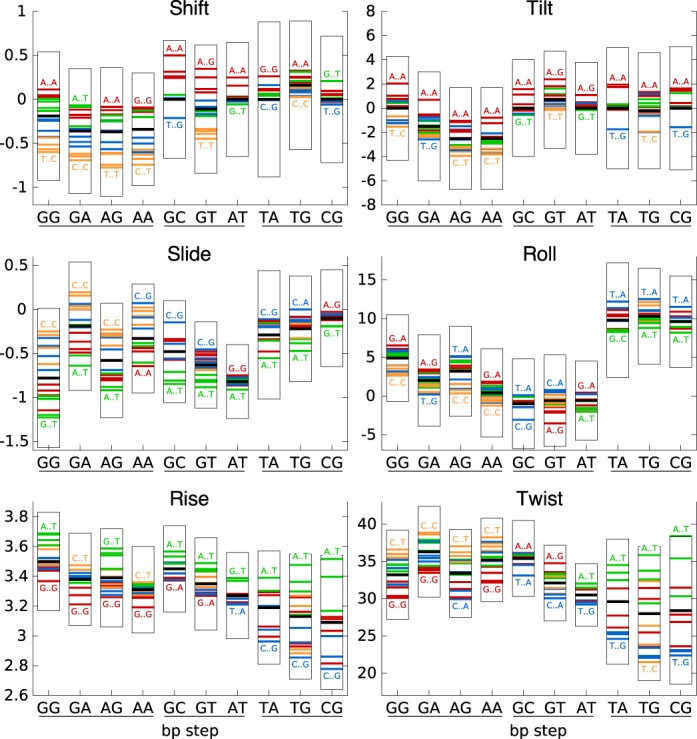
Tetranucleotide sequence effects on inter base pair helical parameter averages. For each parameter, the average value and the standard deviation for the 10 distinct dinucleotide steps (along the abscissa, underlined to show the RR, RY and YR families) are shown by the thick black horizontal lines and surrounding boxes. The impact of the flanking base pair steps on each of these values is shown by the colored horizontal lines: R..R (red), R..Y (green), Y..R (blue), and Y..Y (orange). The extreme values of the averages for each dinucleotide step are indicated by the corresponding flanking sequences.

The results in Figure 1 show how the flanking base pairs can influence the inter-BP parameters of the 10 distinct dinucleotide steps. The first point to note is that, in some cases, the flanking sequence effect for a given dinucleotide step can lead to a range of parameters that is larger than the range covered by the corresponding average values of all the dinucleotide steps taken together. Overall, while tilt and roll seem to be affected rather weakly by the flanking sequence, all the other parameters are sensitive for at least some dinucleotide steps. The influence of the flanking sequence on individual dinucleotide steps is however variable. If we consider all the inter-BP parameters together, it is difficult to see any trends, although the AT step stands out as being very weakly perturbed. For the other steps, the impact depends on the parameter considered: RR steps (and some RY steps) are sensitive in terms of shift and slide, whereas YR steps are sensitive in terms of rise and twist. If we turn to the nature of the flanking sequences (shown by the colored bars in Figure 1), we can certainly see some trends, such as R..Y (green bars) that leads to high rise for all dinucleotide steps, or Y..Y (orange bars) that leads to low rise and twist in YR steps and negative shift and positive slide in RR steps.

**Figure 2.**
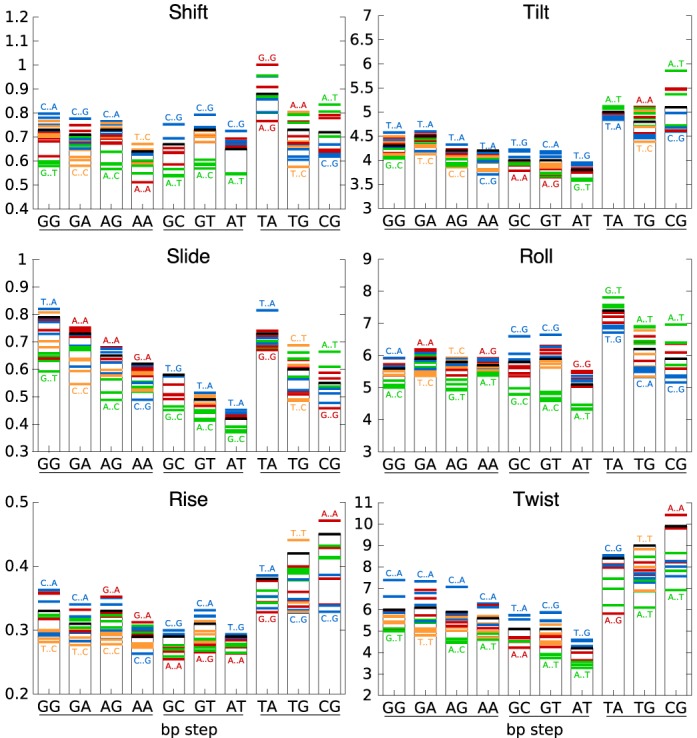
Tetranucleotide sequence effects on inter base pair helical parameter variance. For each of the 10 distinct dinucleotide steps (along the abscissa, underlined to show the RR, RY and YR families), the mean variance is indicated by the thick black horizontal lines and the thin vertical bars. The values for the different families of flanking base pairs are indicated by the colored horizontal lines: R..R (red), R..Y (green), Y..R (blue), and Y..Y (orange). The extreme values of variance for each dinucleotide step are indicated by the corresponding flanking sequences.

Figure 2 shows that the flanking sequence can also have an impact on fluctuations. Once again there are few overall trends, although we can see that dinucleotide steps with high average variance are often also the most affected by the flanking base pairs. This is the case for YR steps that are significantly perturbed by the flanking sequence, particularly in the case of CA and CG for roll, rise and twist, whereas TA is sensitive in terms of shift and twist. However, some parameters show high average variance that is virtually unaffected by the sequence environment, for example, TA slide and roll. We also note that R..Y flanking base pairs tend to reduce the variance of shift, slide and roll for both RR and RY dinucleotide steps.

### Deviations from Gaussian behavior

So far, we have only considered the mean and variance of the helical parameters, which would completely determine their distributions if they were Gaussian. However, as we have seen in earlier work (17,18,24), this is not always the case. Certain base sequences can adopt multiple conformations, resulting in multimodal or, at least, strongly non-Gaussian parameter distributions (for complete histograms of the 50 ns ABC data (24), see the Supplementary Material of (11)). However, determining a measure of the degree of departure from Gaussian behavior is nontrivial. In an earlier study, Dans *et al.* (14) used a Bayesian information analysis to group distributions into different families described as either normal or binormal. However, it is not simple to determine the correct parameters for this analysis, and here we have instead chosen to classify the various distributions by visual inspection.

**Figure 3.**
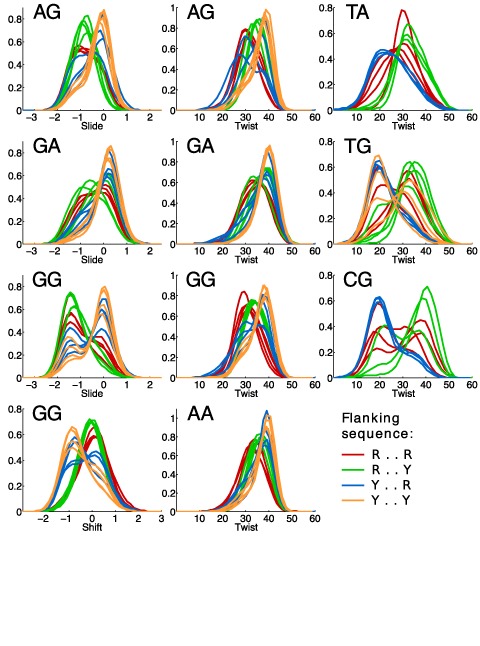
Helical parameter distributions. All inter-BP parameter distributions (shift, slide and twist) showing evident non-Gaussian or multi-peaked behavior. The distributions are grouped according to the central base pair step (all four RR steps appear in the left two columns, and all three distinct YR steps in the right-hand column), and are colored on the basis of the four possible types of flanking sequence (with only three distinct cases for the two self-symmetric dinucleotides).

Figure 3 shows 11 cases of helical parameter distributions that were identified as having significant deviations from Gaussian behavior. Each panel illustrates the distributions of a given helical parameter for all possible flanking sequences of a given dinucleotide step. The 11 panels display all cases for which at least half of the flanking sequence contexts lead to asymmetric distributions, shoulders or multiple peaks. Comparing the different curves within a single panel confirms that the flanking base pairs have a strong effect on the central base pair step parameter distributions. The most extreme example is that of CG twist which suggests that there are at least two wells, one centered at around 20° and the other around 40°. Figure 3 shows that while all CG twist distributions sample both wells, the relative population of the two states is strongly affected by the flanking sequence. In particular Y..R flanking sequences (plotted in blue) lead CG steps to prefer low-twist values, while R..Y flanking sequences (green) favor high twists, and R..R sequences (red) leave the CG step free to sample both states with more uniform frequencies (see also (28)). Similar behaviors can be observed in the other panels of Figure 3 for the twist, shift and slide distributions of the other indicated central dinucleotide steps.

It should be stressed that each distribution plotted in Figure 3 concerns the inter-BP parameters of a single base pair step (within a single tetranucleotide fragment of a given oligomer); therefore, their non-Gaussian, or multi-peaked, character reflects the existence of multiple substates and not a sequence-averaged behavior. This behavior is therefore distinct from the well-known property of certain base pair steps (notably YR steps, see, for example (44,45)) to adopt different conformations within different sequence contexts. Several examples of the underlying conformational transitions we observe are illustrated with helical parameter time series in Supplementary Figure S5. It is interesting to note that our finding has found experimental support in recent high-resolution X-ray structures that indeed reveal multiple conformations of the B-DNA oligomer CCAGGCCTGG within a single crystal involving both the backbone and helical parameters (notably slide) (46).

**Figure 4.**
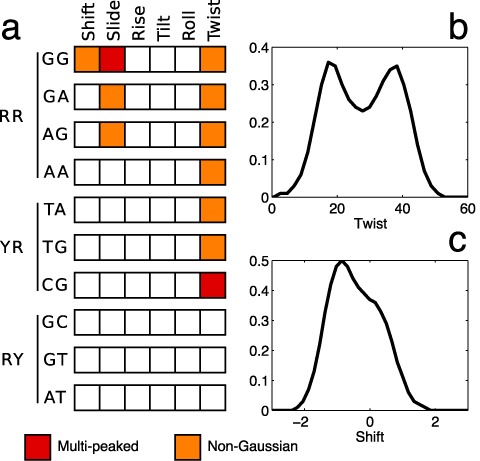
Non-Gaussian and multi-peaked helical parameter distributions. (**a**) Probability distributions of the inter-BP parameters for the central base pair step of the 136 distinct tetranucleotide sequences were inspected for visible deviations from Gaussian behavior. Helical parameters classed as ‘Multi-peaked’ (red) have two distinct peaks in their distributions for most flanking sequences. Monomodal distributions with obvious deviations from normality (such as pronounced shoulders or asymmetry) for most flanking sequences are classed as ‘Non-Gaussian’ (orange). The results are grouped on the basis of the purine/pyrimidine family of the dinucleotide step. Examples of parameter distributions (see also Figure 3) are shown for the twist of AGCA ((**b**), multi-peaked) and the shift of TGGT ((**c**), non-Gaussian).

The conclusions of our analysis are summarized in Figure 4 and can be compared with the results of Dans *et al.* (14) in Supplementary Figure S6. The results are: (i) strongly non-Gaussian distributions do not arise at all in RY steps, and are restricted to shift for GG steps, slide for all RR steps, and twist for all RR and YR steps. The other three inter-BP, as well as all intra-BP, parameters do not significantly deviate from Gaussian behavior in any sequence context; (ii) only GG slide and CG twist show clear multiple peaks. We remark that while our analysis has identified tetranucleotide fragments whose central base pair steps interconvert between multiple conformational substates, it certainly does not rule out the possibility that other fragments can also have multiple substates, but with populations that are difficult to distinguish using only helical parameter distributions.

### Tetranucleotide sequence effects on backbone conformations

**Figure 5.**
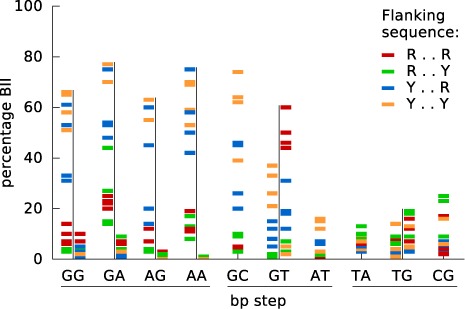
Sequence dependence of BII backbone conformations. The percentage occurrence of BII backbone states for the phosphodiester junction of each of the 10 distinct base pair steps is shown. For each step, the results for the Watson and Crick strands are plotted as colored bars on the left and right of the vertical black line (for self-complementary steps, GC, AT, TA and CG, the two strands are indistinguishable and only one column of results is plotted). Each bar refers to one of the 136 distinct tetranucleotide fragments, colored according to its sequence on the Watson strand.

We can now turn to an analysis of DNA backbone parameters and, in particular, to the ϵ (C3′-O3′) and ζ (O3′-P) dihedrals that have already been shown to play an important role in defining B-DNA conformational substates (24,47–49). The reason for this is that backbone dihedrals preferably occupy distinct conformations, gauche^+^ (g+), gauche^−^ (g–) or trans (t), which represent minima of the corresponding torsional energy. In the case of ϵ and ζ, which lie on the 3′-side of each deoxyribose sugar, transitions tend to be coupled and favor the combinations (ϵ = t)/(ζ = g–), known as BI and characteristic of canonical B-DNA, or (ϵ = g–)/(ζ = t), known as BII. The base stacking preferences of a given dinucleotide step can favor a BI/BII transition, leading to a discrete conformational substate reflected not only in the phosphodiester backbone, but also in the local helical parameters. In fact, not only the nature of the dinucleotide steps, but also the nature of their flanking sequences strongly influence the proportion of neighboring BI and BII conformations. This is illustrated in Figure 5 (see also Supplementary Figure S7 for a more detailed view of the BI/BII distributions). As we can see, the percentage occurrence of BII states is very variable. Thus, YR steps (irrespective of the flanking sequence) strongly disfavor BI–BII transitions and the BII population rarely exceeds 20% in either strand. This is also the case for AT steps and for the Crick strand of all RR steps. In contrast, the Watson strand of RR steps, and both strands of GC and GT steps show highly variable BII percentages that are strongly influenced by the flanking base pairs and, in particular, by the base pair on the 5′-side. Looking at the color-coded results in Figure 5, we can see that for each of the seven RR and RY steps, a 5′-flanking pyrimidine (blue Y..R and orange Y..Y families) favors relatively high BII percentages, while low percentages occur with a 5′-purine (R..R and R..Y shown in red and green, respectively). Note that this is also true for the Crick strand of GT steps, where the R..R flanking sequence (red) on the Watson strand corresponds to Y..Y on the Crick strand.

**Figure 6.**
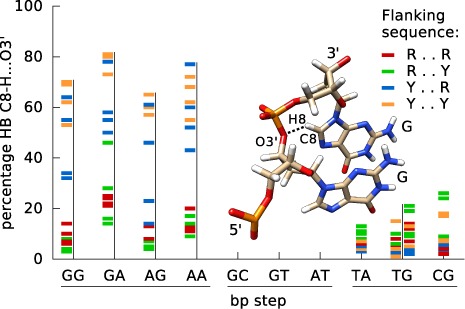
Sequence-dependent formation of C8-H…O3′ hydrogen bonds. The percentage occurrence of C8-H…O3′ hydrogen bonds involving a 3′-purine and the junction phosphate of the 10 distinct dinucleotide steps is shown. For each step, the results for the Watson and Crick strands are plotted as colored bars on the left and right of the vertical black line (for self-complementary steps, GC, AT, TA and CG, the two strands are indistinguishable and only one column of results is plotted). Each bar refers to one of the 136 distinct tetranucleotide fragments, colored according to its sequence on the Watson strand. Note that 3′-pyrimidines (and thus all RY steps) cannot form this hydrogen bond. The inset is a stick representation of a GG base pair step showing the atoms involved in the formation of the C8-H…O3′ hydrogen bond.

Recent work by Dans *et al.* (28) on the polymorphism of the CG step observed that BII states in the GR step of a CGR trinucleotide are associated with the formation of a C8-H…O3′ hydrogen bond between the C8-H group of the R base and the O3′ atom of the corresponding 5′-phosphate group. We can now extend the Dans *et al.* (28) analysis to the full set of sequences in the ABC microsecond trajectories. Inspecting the time series and the overall distribution of C8…O3′ distances shows that a 4-Å cutoff is appropriate to separate bonded and unbonded states. The resulting tetranucleotide-dependent occupancies for the central C8-H…O3′ hydrogen-bonded state (which cannot arise for central RY steps) are summarized in Figure 6. For central RR steps, there is a perfect correlation between the occupancies of the hydrogen-bonded state (Figure 6) and the BII state (Figure 5) for all tetranucleotide contexts. An almost identical correlation is observed for YR steps. A more detailed analysis of the associated time series shows that, averaging over all cases with central RR and YR dinucleotides, BII conformations are associated with backbone hydrogen bonds in 90% of the snapshots and, conversely, backbone hydrogen bonds are associated with BII conformations in 87% of snapshots. This very high correlation holds for both high (generally central RR) and low (generally central YR) BII occupancy steps.

### Relating inter-base pair helical parameters with backbone torsions

**Figure 7.**
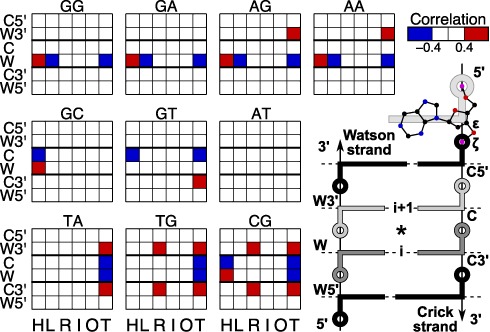
Partial cross correlation matrices between helical parameters and the ζ (O3′-P) backbone torsion. Cross correlations of the inter-BP parameters, shift (H), slide (L), rise (R), tilt (I), roll (O) and twist (T) and backbone torsion ζ are shown at three consecutive levels, grouped according to the central dinucleotide step. Positive correlations >0.4 are shown in red and negative correlations <–0.4 are shown in blue. The schematic representation of a double-stranded tetranucleotide fragment on the right defines the naming convention for the ζ torsions and the six backbone segments: inter-BP parameters refer to the central junction (*) and are grouped according to the dinucleotide sequence at levels i and i+1 on the Watson strand.

We will now take a further step in understanding how BI–BII backbone transitions are coupled to changes in helical parameters. Either of the ϵ or ζ backbone torsions (or the difference ϵ-ζ (47)) could be used for this purpose, but here we limit the analysis to the ζ (O3′-P) torsion. Figure 7 shows that there are many strong (both positive and negative) correlations between ζ and inter-BP parameters (averaged over the flanking sequences). The correlations are highly dependent on the base pair step considered, although the parameters affected are limited to shift, slide and twist (with two exceptions involving rise). Depending on the base pair step, the coupling with helical parameters can involve the conformation of any one of six backbone segments, namely W5′, W, W3′, C5′, C, and C3′, as defined in Figure 7. RR steps (top row of Figure 7) show a distinctive pattern of correlations involving shift, slide and twist with the Watson backbone of the central junction. Note that the sign of the correlations suggests that slide and twist will be positively correlated with one another and negatively correlated with shift. YR steps (bottom row) show correlations between twist and both strands of the central backbone junction, but also between rise and twist and the 3′-junctions in both strands (W3′ and C3′). RY steps (middle row) have more heterogeneous properties, but it is notable that AT steps, whose conformations were the least influenced by the flanking base pairs (see Figure 1), are also the only steps that show no strong correlation with backbone conformations.

### An overview of sequence-dependent effects on B-DNA conformation

**Figure 8.**
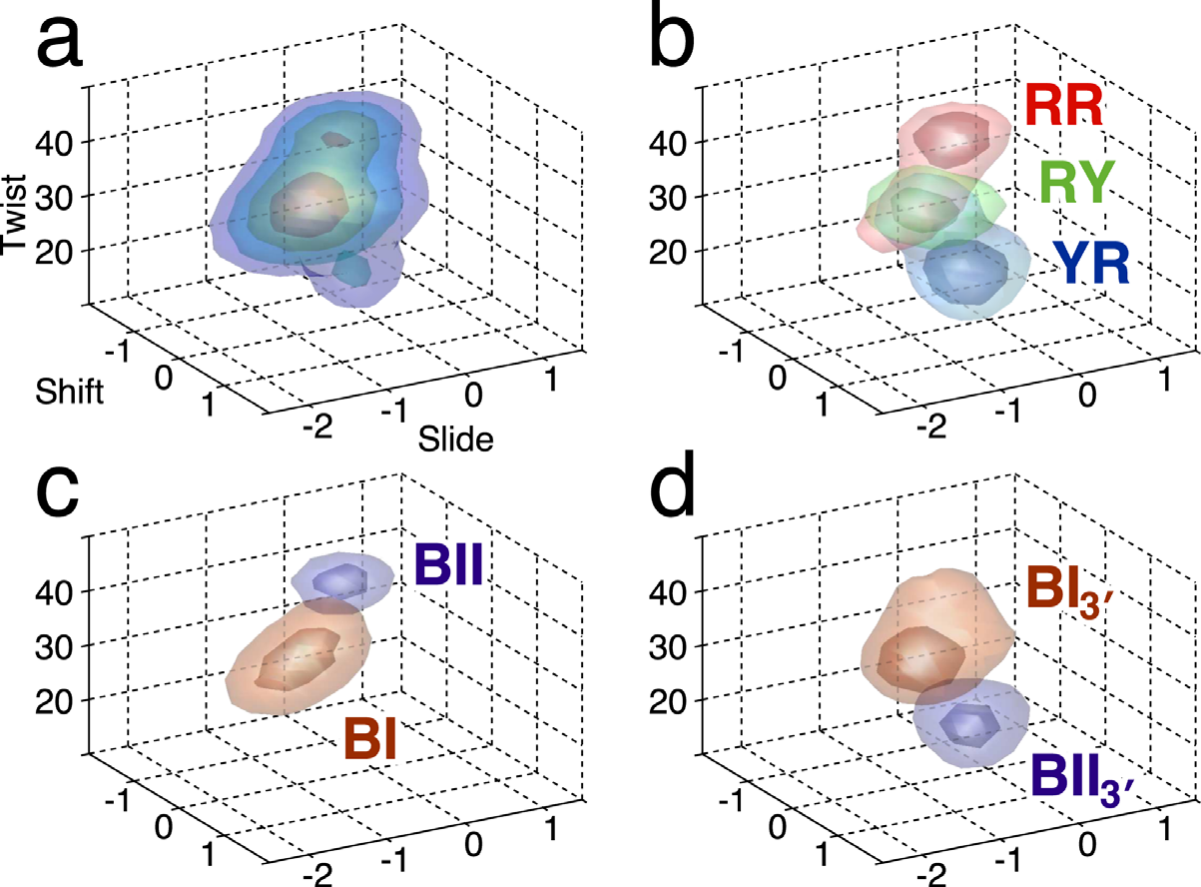
Probability density as a function of shift, slide and twist. (**a**) Probability density isosurfaces (generated from uniform bin histograms) shown at four evenly spaced levels as a function of the inter-BP parameters shift, slide and twist averaged over all tetranucleotides. The surfaces enclose the most densely populated region of this conformational space, i.e. the peaks of the distribution (namely, 15%, 30%, 45% and 60% of the maximum density). (**b**) Same as (a), but dividing the probability density into RR (red), RY (green) and YR (blue) sequences for the corresponding base pair step (see the main text for the precise definition of the RY and YR groups). In this case, only the two innermost isodensity surfaces are shown for each sequence group. (**c**) RR steps are further subdivided according to the BI/BII conformational state of the Watson backbone in the base pair step. (**d**) YR steps are further subdivided according to the conformational state of the 3′-junctions: BI_3′_ indicates that both 3′-junctions (W3′ and C3′ in Figure 7) are in the BI state, and BII_3′_ indicates that at least one of these junctions is in the BII state.

In light of what we have seen in terms of non-Gaussian parameter distributions, backbone substates and the link between backbone and helical parameters, we can now attempt to build a concise description of how base sequence is linked to B-DNA conformation. Given that non-Gaussian parameter distributions are limited to the inter-BP parameters shift, slide and twist and that these parameters also show the highest correlations with the backbone conformations, we can start by representing the ensemble of the conformations observed for a set of all 136 distinct tetranucleotide sequences as a probability density plot in the space of these three parameters. As discussed in the methodology section, the sign of four helical variables (shift, tilt, shear and buckle) depends on the choice of the naming convention that sets the ‘Watson’ strand in each dinucleotide. Since the sign of shift is important in the analysis that follows, we will use the average shift of the central dinucleotide of each tetranucleotide to guide our choice of names used for each of the distinct cases. By identifying the dinucleotides that have negative average shift (GG, GA, AG, AA, GT, CA), we define a direction in which all non-self-complementary tetranucleotides are read. Note that self-complementary dinucleotides (AT, TA, CG, GC) have average shift values close to zero due to their inherent symmetry. The results presented in Figure 8 (3-dimensional probability densities) and Supplementary Figure S8 (2-dimensional projections of the results in Figure 8) respect this convention.

The isodensity surfaces in Figure 8a show that there are three distinct regions of high density (i.e. peaks of the probability distribution) that differ primarily in terms of their helical twist, and reflect the existence of three distinct conformational substates. The most populated (large orange) state is centered at the most canonical values of twist and shift, for B-DNA, but has negative values of slide. The second (small orange) state is centered at higher twist (approximately +5°) and has negative shift with slide close to zero. The last (small blue) state occurs at very low twist (approximately −15°) and has shift close to zero with a small negative slide. In fact, these three states can also be distinguished in terms of base sequence at the purine–pyrimidine level, as shown in Figure 8b. While the most populated state involves all base pair steps, the high and low-twist regions are mainly occupied by RR and YR steps, respectively, and correspond to the highest probability densities for these two sequence families. These observations are compatible with the data shown in Figure 3.

Comparison of these results with the backbone-helical parameter correlation analysis presented earlier allows us to shed light on how the populations of the three B-DNA conformational substates are linked to backbone conformation and, because of their high correlation, also to the formation of the C8-H…O3′ hydrogen bond in RR steps. First, note that the transition to the high-twist state for RR steps involves coupled changes in shift and slide, and these three parameters have indeed been shown to strongly correlate with the backbone torsion ζ of the Watson strand of RR steps. The isodensity surface shown in Figure 8c confirms that BI–BII transitions in the Watson strand of RR steps are indeed concerted with the changes in shift, slide and twist and lead to two substates with clearly distinguished probability density clouds corresponding to the high-twist (BII) and intermediate-twist (BI) states.

Populating the low-twist state for YR steps is similarly coupled to BI–BII transitions, but in this case the transitions concern the two 3′-flanking junctions (denoted as W3′ and C3′ in Figure 7), a behavior already observed in the particular case of CG steps in the Dickerson dodecamer (22). The low-twist state is occupied when either or both of these junctions are in the BII state as shown in Figure 8d. In contrast to the high-twist state seen with RR steps, the low-twist state does not involve significant changes in shift or slide, in agreement with the absence of cross correlations between the backbone and these variables for YR steps (see Figure 7). Note that although the twist of YR steps is also correlated with the backbone conformation of these steps, this may be due mainly to the negative correlation of BI/BII states typically seen at adjacent steps within a given backbone. Note also that, at least for TG and CG steps, transitions to the low-twist state are correlated with significant reductions in rise as expected from the results in Figure 7.

Lastly, RY steps, which show the sparsest correlations with the backbones (see Figure 7) and do not exhibit non-Gaussian behavior (see Figure 4), are largely restricted to the single canonical probability density region with intermediate twist values (see Figure 8b).

We have identified three conformational states of B-DNA that differ in particular with respect to their values of shift, slide and twist, and their backbone conformations, as well as clear-cut dinucleotide preferences for occupying just one or two of these states. Furthermore, while RR steps are largely restricted to the high- and intermediate-twist states, we do observe YR steps also occupying twist values above 35° with significant probability (see Figure 3, right column, and the BI_3′_ probability in Figure 8d and Supplementary Figure S8d). This observation suggests that some flanking sequences induce an exceptional behavior in TG and especially CG steps, which deserves further investigation. Finally, we remark that defining the number of states is ultimately a subjective decision, and our results do not exclude the existence of finer subdivisions, e.g. multiple substates within the intermediate-twist basin.

Given that we generally observe anticorrelation between the BI/BII states (within a given strand), and also the twist, of adjacent base pair steps, we can expect to see some structural coupling beyond the dinucleotide level. We can make two preliminary steps in this direction. First, for trinucleotides, we note that Figure 5 shows high BII occupancies for RR and RY steps when these steps are preceded by a pyrimidine (i.e. YRR or YRY). Indeed, backbones with at least 30% of BII belong to the second junction of the bimodal YRX trinucleotides (see Figure 8d) in all but one case.

Second, by combining the individual tendencies of three overlapping dinucleotides, we can predict some tetranucleotide structural properties shown in Figure 3. As an example, consider the TG twist distributions (third column, second panel from the top in Figure 3), and, in particular, the YTGR tetranucleotides (blue curves). The final GR step can populate the high-twist RR state, as can the first YT step (which is RR on the Crick strand): the presence of two flanking RR, potentially high-twist, steps then explains the observed preference for low twist of the central TG step. Furthermore, high twist in the two flanking steps correlates with BII backbone states on the Watson strand for the GR step, and on the Crick strand for the YT step. These backbones correspond to the W3′ and C3′ backbones with respect to the TG step, explaining the correlation we observe between TG twist and the backbone state of the flanking steps. On the other hand, RTGR and YTGY tetranucleotides (red and orange curves) have flanking RR and RY steps. Since RY steps mainly populate the intermediate-twist state, we observe weaker selectivity for the low-twist state of TG (and more pronounced polymorphism) in both RTGR and YTGY. Correspondingly, RTGY tetranucleotides, with two flanking RY steps, show high selectivity for the intermediate-twist state of TG.

## CONCLUSION

The results of well-converged, microsecond-scale molecular dynamics trajectories on 39 oligomers enable us to formulate a clearer view of how base sequence influences the structure and dynamics of double-helical B-DNA. First, it is shown that B-DNA undergoes very large fluctuations on this timescale, including the transitory formation of sharp kinks toward the major and minor grooves, the spontaneous creation of short lived, potential intercalation sites and transient base pair opening, not only at the ends of the oligomers, but also in their centers. Second, it is shown that average base pair step helical parameters and their fluctuations can be influenced more strongly by the base pairs flanking the step (i.e. the tetranucleotide sequence) than by the nature of the base pair step itself. Third, many tetranucleotide sequences exhibit oscillations between multiple conformational substates. These substates are notably characterized by multimodal distributions of the helical parameters shift, slide and twist. The existence of these substates is linked to BI/BII transitions in the phosphodiester backbones. We also observe that within RR and YR steps a BII backbone conformation is highly correlated with the formation of base-phosphate (C8-H…O3′) hydrogen bonds.

Putting these observations together starts to provide a clearer overview of the conformational space occupied by B-DNA as a function of its base sequence. This space can be divided into three main regions in terms of shift, slide and twist. The first region is characteristic of canonical B-DNA and can be occupied by all sequences. The second is characterized by higher twist and negative shift and is largely restricted to purine–purine (RR) steps. The last region is characterized by very low twist and negative slide and is mainly populated by pyrimidine–purine (YR) steps. Thus both RR and YR steps can occupy two distinct conformational regions and can also exhibit multimodal behavior in a single sequence context. In addition, because YR steps are sensitive to the state of neighboring 3′ backbones further coupling beyond the dinucleotide sequence may occur in longer fragments, e.g. in YRX trinucleotides.

Analysis of our data set is a significant step toward achieving a comprehensive view of the sequence-dependent behavior of B-DNA and constitutes a valuable resource for the further development of coarse-grain models of DNA. By carrying out microsecond simulations we are approaching biologically relevant timescales. These results constitute a more thorough sampling of conformational space, for a broader range of sequences, than has been possible before. Most importantly, we begin to see that the surprisingly large impact of base sequence on the double helix can be understood in relatively simple terms.

## SUPPLEMENTARY DATA

Supplementary Data are available at NAR Online.

SUPPLEMENTARY DATA
