# Supplementary material for: μABC: a systematic microsecond molecular dynamics study of tetranucleotide sequence effects in B-DNA
Source: Nucleic Acids Res. 2014 Sep 26;42(19):12272–83. doi: 10.1093/nar/gku855 (PMC4231739; doi:10.1093/nar/gku855)
Supplement: SUPPLEMENTARY DATA [file supp_42_19_12272__index.html]

μABC: a systematic microsecond molecular dynamics study of tetranucleotide sequence effects in B-DNA — μABC: a systematic microsecond molecular dynamics study of tetranucleotide sequence effects in B-DNA — SUPPLEMENTARY DATA 

# μABC: a systematic microsecond molecular dynamics study of tetranucleotide sequence effects in B-DNA

## SUPPLEMENTARY DATA

**Files in this Data Supplement:**

- SUPPLEMENTARY DATA
