# Supplementary material for: μABC: a systematic microsecond molecular dynamics study of tetranucleotide sequence effects in B-DNA
Source: Nucleic Acids Res. 2014 Sep 26;42(19):12272–83. doi: 10.1093/nar/gku855 (PMC4231739; doi:10.1093/nar/gku855)
Supplement: SUPPLEMENTARY DATA [file supp_gku855_microABC_supporting_information.pdf]

SUPPORTING INFORMATION

Nucleic Acids Research, 2014

doi: *10.1093/nar/gku855*

µABC: a systematic microsecond molecular dynamics study of tetranucleotide sequence effects in B-DNA

Marco Pasi[1], John H. Maddocks[1,*], David Beveridge[2], Thomas C. Bishop[3], David A. Case[4], Thomas Cheatham III[5], Pablo D. Dans[6], B. Jayaram[7], Filip Lankas[8], Charles Laughton[9], Jonathan Mitchell[1], Roman Osman[10], Modesto Orozco[6], Alberto Pérez[6], Daiva Petkevičiūtė[1], Nada Spackova[11], Jiri Sponer[11,12], Krystyna Zakrzewska[13] and Richard Lavery[13,*]

[1] Section de Mathématiques, Swiss Federal Institute of Technology (EPFL), CH-1015 Lausanne, Switzerland
[2] Department of Chemistry, Wesleyan University, Middletown, CT 06459, USA
[3] Departments of Chemistry & Physics, Louisiana Tech University, Ruston, LA 71270, USA
[4] BioMaPS Institute and Dept. of Chemistry & Chemical Biology, Rutgers University, 610 Taylor Road, Piscataway, NJ 08854-8087, USA
[5] Department of Medicinal Chemistry, University of Utah, Skaggs 307, Salt Lake City UT. 84112, USA
[6] Joint BSC-CRG-IRB Program on Computational Biology, Institute of Research in Biomedicine, Parc Científic de Barcelona, Josep Samitier 1-5, Barcelona 08028. Spain and Barcelona Supercomputing Centre, Jordi Girona 31, Edifici Torre Girona. Barcelona 08034, and Departament de Bioquímica, Facultat de Biología, Avgda Diagonal 647, Barcelona 08028, Spain
[7] Department of Chemistry, Indian Institute of Technology, Hauz Khas, New Delhi 110016, India
[8] Institute of Organic Chemistry and Biochemistry, Academy of Sciences of the Czech Republic, Flemingovo nam. 2, 166 10 Praha 6, Czech Republic
[9] School of Pharmacy and Centre for Biomolecular Sciences, University of Nottingham, NG7 2RD, UK
[10] Department of Structural and Chemical Biology, Mount Sinai School of Medicine, New York, NY 10029, USA
[11] Institute of Biophysics, Academy of Sciences of the Czech Republic, Kralovopolska 135, 612 65 Brno, Czech Republic
[12] CEITEC - Central European Institute of Technology, Masaryk University, Campus Bohunice, Kamenice 5, 625 00 Brno, Czech Republic
[13] Bases Moléculaires et Structurales des Systèmes Infectieux, CNRS UMR 5086 / Univ. Lyon I, IBCP, 7 Passage du Vercors, 69367 Lyon, France

*To whom correspondence should be addressed. Email: richard.lavery@ibcp.fr; Tel: +33 4 72 72 26 37; Fax +33 4 72 72 26 04. Email: john.maddocks@epfl.ch; Tel: +41 21 693 27 62; Fax: +41 21 693 55 30.

Table S1. The 39 18-base pair oligomers studied in this work, showing the unique tetranucleotides constituting the repeating sequence of each oligomer.

Name	Sequence	Tetranucleotides
AAAA	GCAAAAAAAAAAAAAAGC	AAAA
AAAC	GCACAAACAAACAAACGC	AAAC, AACA, ACAA, CAAA
AAAT	GCATAAATAAATAAATGC	AAAT, AATA, ATAA, TAAA
AGAG	GCAGAGAGAGAGAGAGGC	AGAG, GAGA
AGCG	GCCGAGCGAGCGAGCGGC	AGCG, GCGA, CGAG, GAGC
AGCT	GCCTAGCTAGCTAGCTGC	AGCT, GCTA, CTAG
AGGA	GCGAAGGAAGGAAGGAGC	AGGA, GGAA, GAAG, AAGG
AGGC	GCGCAGGCAGGCAGGCGC	AGGC, GGCA, GCAG, CAGG
AGGT	GCGTAGGTAGGTAGGTGC	AGGT, GGTA, GTAG, TAGG
AGTC	GCTCAGTCAGTCAGTCGC	AGTC, GTCA, TCAG, CAGT
AGTG	GCTGAGTGAGTGAGTGGC	AGTG, GTGA, TGAG, GAGT
ATGC	GCGCATGCATGCATGCGC	ATGC, TGCA, CATG
CAAG	GCAGCAAGCAAGCAAGGC	CAAG, AAGC, AGCA, GCAA
CAAT	GCATCAATCAATCAATGC	CAAT, AATC, ATCA, TCAA
CGCG	GCCGCGCGCGCGCGCGGC	CGCG, GCGC
CGGA	GCGACGGACGGACGGAGC	CGGA, GGAC, GACG, ACGG
CGGC	GCGCCGGCCGGCCGGCGC	CGGC, GGCC, CCGG
CGGT	GCGTCGGTCGGTCGGTGC	CGGT, GGTC, GTCG, TCGG
CGTA	GCTACGTACGTACGTAGC	CGTA, GTAC, ACGT
CGTG	GCTGCGTGCGTGCGTGGC	CGTG, GTGC, TGCG, GCGT
GAAA	GCAAGAAAGAAAGAAAGC	GAAA, AAAG, AAGA, AGAA
GAAC	GCACGAACGAACGAACGC	GAAC, AACG, ACGA, CGAA
GAAT	GCATGAATGAATGAATGC	GAAT, AATG, ATGA, TGAA
GATA	GCTAGATAGATAGATAGC	GATA, ATAG, TAGA, AGAT
GGGA	GCGAGGGAGGGAGGGAGC	GGGA, GGAG, GAGG, AGGG

Name	Sequence	Tetranucleotides
GGGC	GCGCGGGCGGGCGGGCGC	GGGC, GGCG, GCGG, CGGG
GGGG	GCGGGGGGGGGGGGGGGC	GGGG
GGGT	GCGTGGGTGGGTGGGTGC	GGGT, GGTG, GTGG, TGGG
TAAC	GCACTAACTAACTAACGC	TAAC, AACT, ACTA, CTAA
TAAG	GCAGTAAGTAAGTAAGGC	TAAG, AAGT, AGTA, GTAA
TAAT	GCATTAATTAATTAATGC	TAAT, AATT, TTAA
TATA	GCTATATATATATATAGC	TATA, ATAT
TCGA	GCGATCGATCGATCGAGC	TCGA, CGAT, GATC
TGGA	GCGATGGATGGATGGAGC	TGGA, GGAT, GATG, ATGG
TGGC	GCGCTGGCTGGCTGGCGC	TGGC, GGCT, GCTG, CTGG
TGGT	GCGTTGGTTGGTTGGTGC	TGGT, GGTT, GTTG, TTGG
TGTA	GCTATGTATGTATGTAGC	TGTA, GTAT, TATG, ATGT
TGTC	GCTCTGTCTGTCTGTCGC	TGTC, GTCT, TCTG, CTGT
TGTG	GCTGTGTGTGTGTGTGGC	TGTG, GTGT

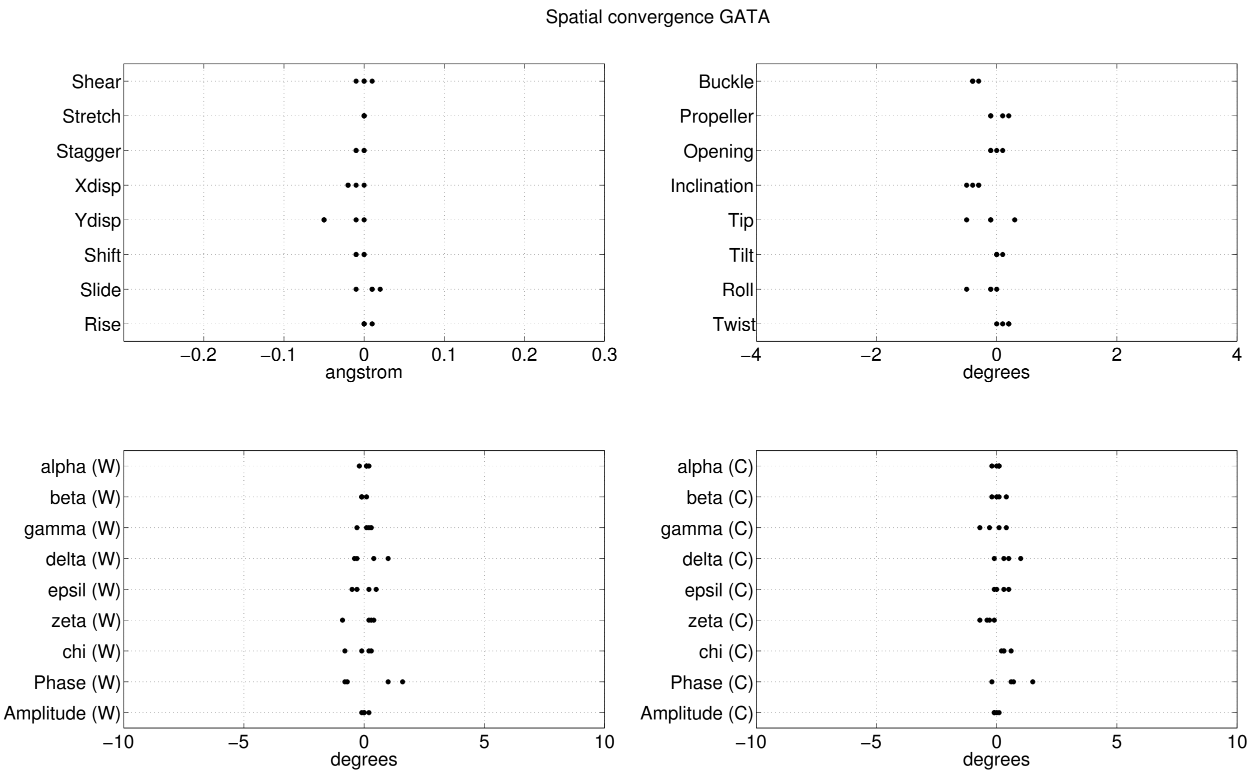

Figure S1: Conformational convergence of µABC simulations. Spatial convergence of the simulations for a chosen ABC oligomer was assessed by comparing average helical and backbone parameters within two occurrences of the same tetranucleotide sequence. The two tetranucleotides were chosen at positions 6-9 and 10-13, towards the center of the oligomer. The differences of the averages are plotted as black dots for each base pair or base pair step within the tetranucleotides. This figure shows the results for the GATA oligomer, which was amongst those with the best conformational convergence properties.

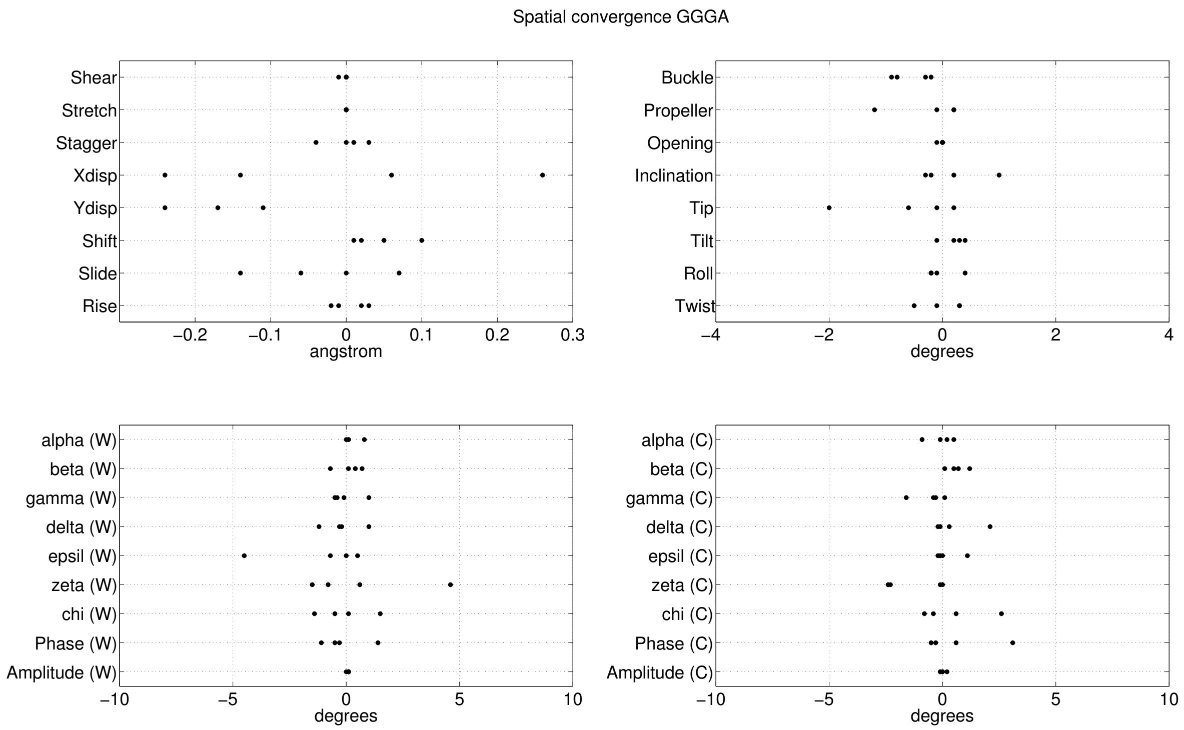

Figure S2: Conformational convergence of μABC simulations. Results for the GGGA oligomer, which was amongst those with the worst conformational convergence properties (see figure S1 for details)

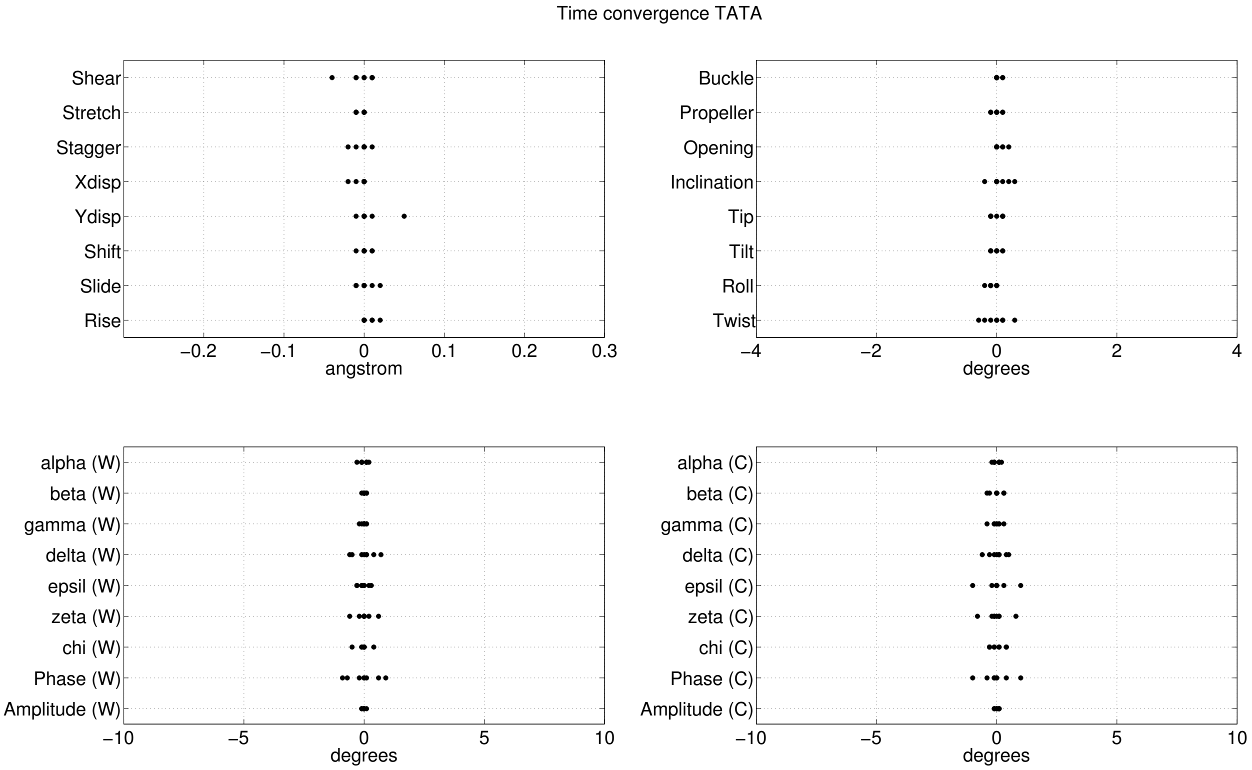

Figure S3: Time convergence of μABC simulations. Time convergence of simulations for a chosen ABC oligomer was assessed by comparing average helical and backbone parameters calculated using either the first half (H_1) or the second half (H_2) of the corresponding trajectory with the averages obtained for the entire trajectory (T). The differences $T–H_1$ and $T–H_2$ are plotted as black dots for each base pair or base pair step within the central tetranucleotide (positions 7-10). This figure shows the results for the TATA oligomer, which was amongst those with the best time convergence properties.

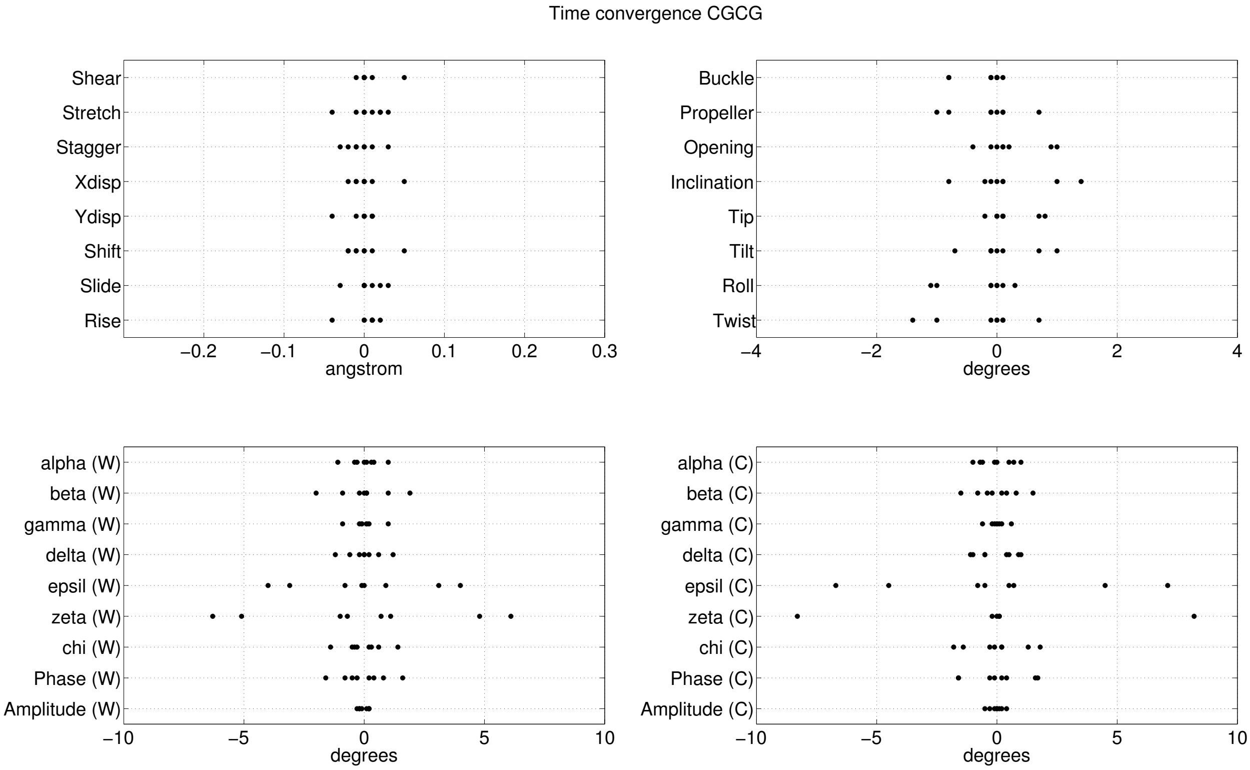

Figure S4: Time convergence of µABC simulations. Results for the CGCG oligomer, which was amongst those with the worst time convergence properties (see Figure S3 for details).

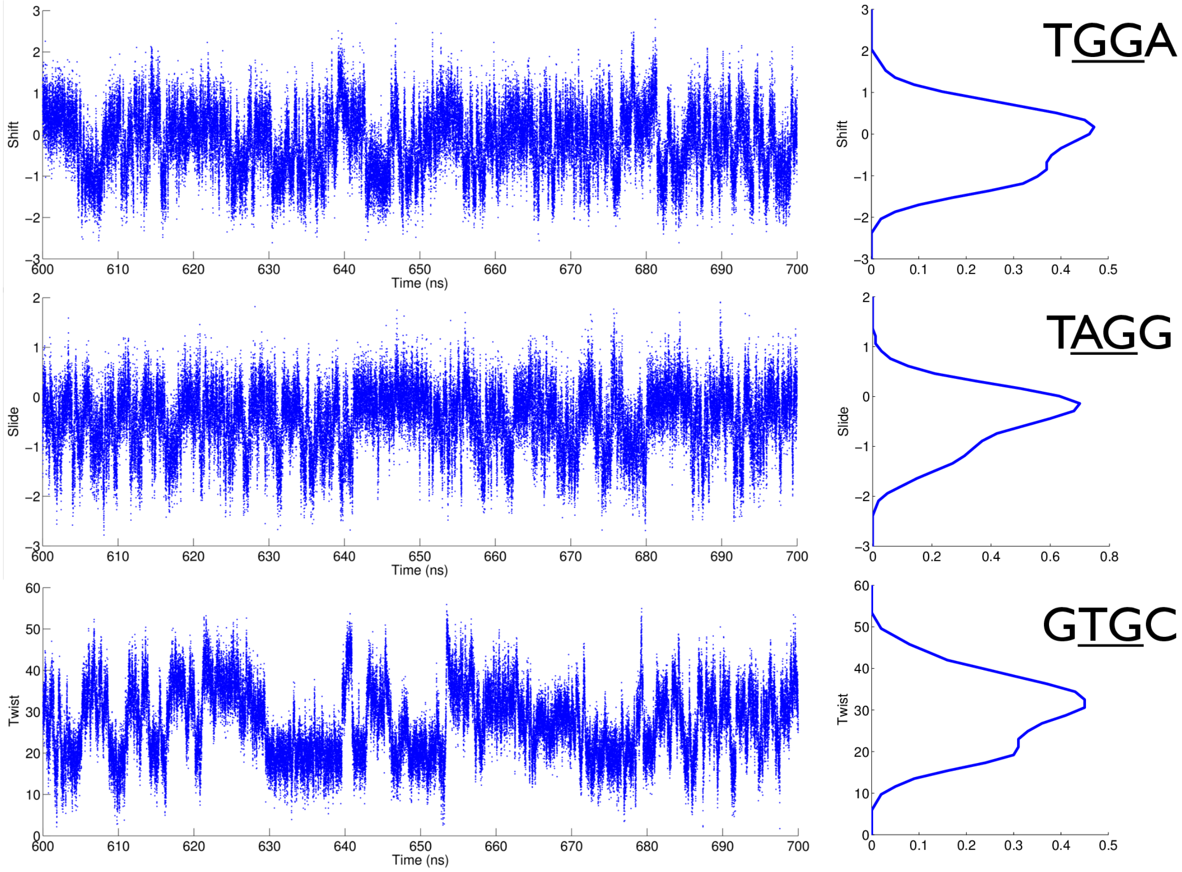

Figure S5: Evidence of conformational transitions. Three examples of inter-BP parameter time series (with the corresponding time-averaged distributions) showing transitions between at least two conformational substates: TGGA shift (top), TAGG slide (middle); GTGC twist (bottom). The parameters refer to the tetranucleotide closest to the center of each oligonucleotide and, for clarity, are limited to a 100 ns interval extracted after 600 ns of simulation.

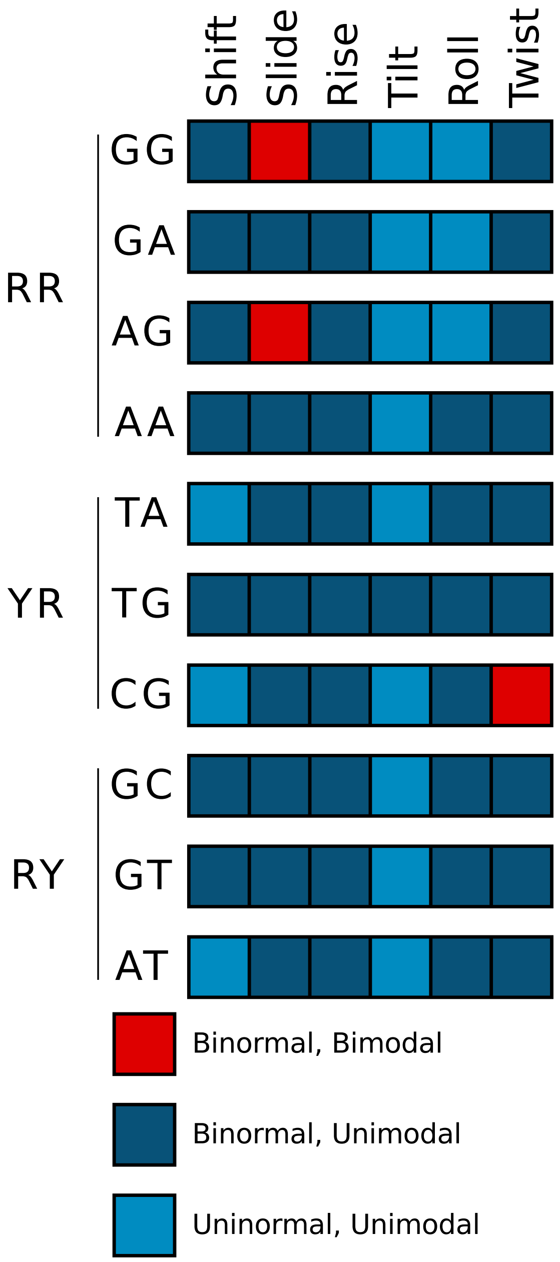

Figure S6: Deviations from Gaussianity in helical parameter distributions. This presentation has been adapted from results in by Dans *et al.* (*Nucleic Acids Res.* 2012 40:10668-10678) for ease of comparison with the data shown in Figure 4 of the main text.

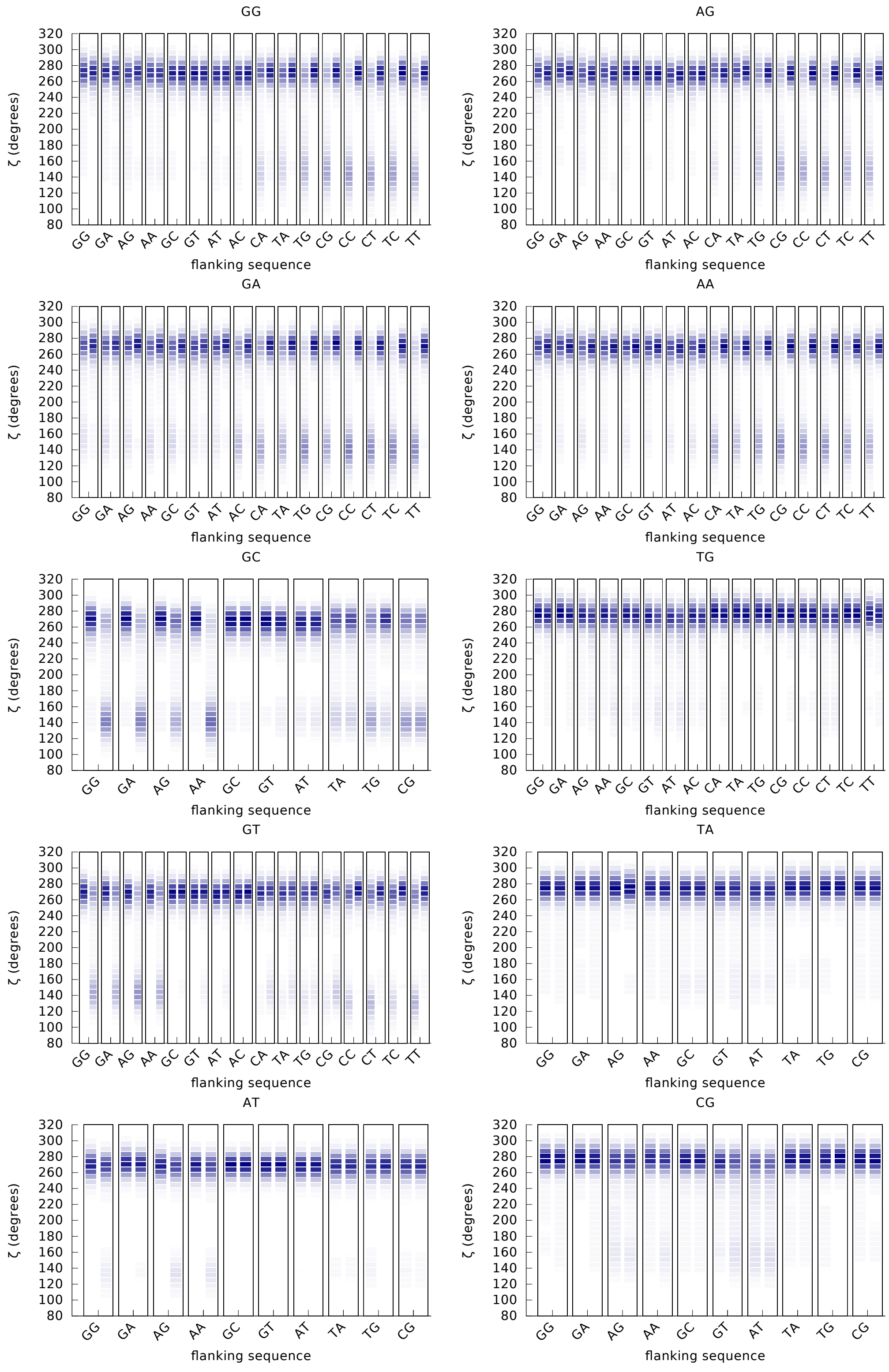

Figure S7: Tetranucleotide sequence effects on backbone dihedral angle ζ. The distribution of the backbone dihedral angle ζ is computed for both strands of the central base pair step of each of the 136 unique tetranucleotide sequences. Distributions are shown as histograms for the Watson and Crick strands (left and right column respectively inside the black frames), where each small rectangle represents a 6.5° bin, and the color intensity is proportional to the BII population. For each central base pair step (one per panel), either 16 pairs or (for self-complementary steps) 10 pairs of histograms are plotted grouped according to flanking sequence X..Y (shown along the abscissa).

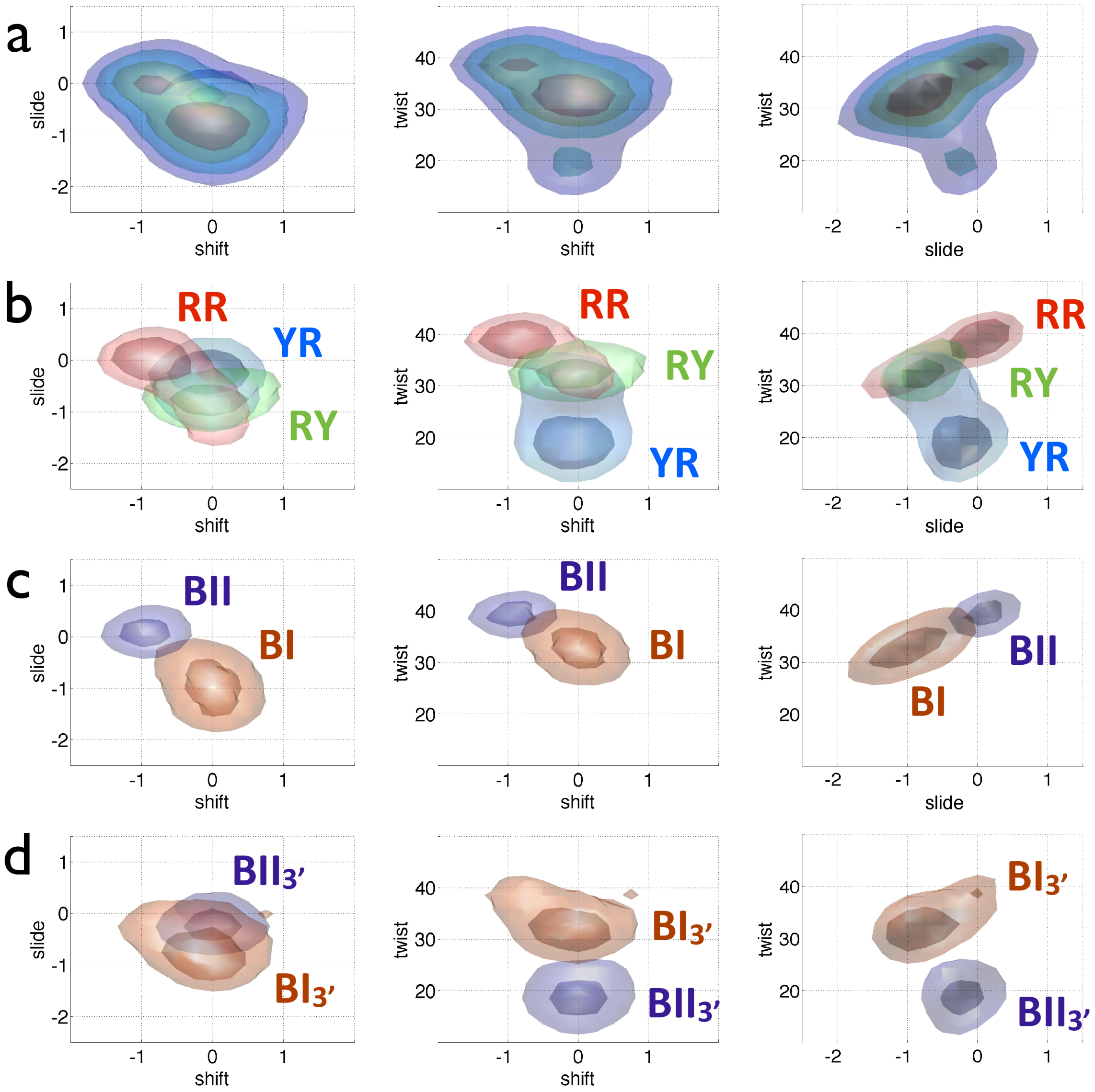

Figure S8: Two-dimensional projections of shift-slide-twist probability distributions. Two-dimensional projections shift-slide (left column), shift-twist (middle column), and slide-twist (right column) of the four probability density plots shown in figure 8: the rows from top to bottom are the three 2D projections of, respectively, panels a to d.
